# Human iPSC Reprogramming Success: The Impact of Approaches and Source Materials

**DOI:** 10.1155/sci/2223645

**Published:** 2025-01-16

**Authors:** Tatyana Pozner, Christine Grandizio, Matthew W. Mitchell, Nahid Turan, Laura Scheinfeldt

**Affiliations:** Biobanking Department, Coriell Institute for Medical Research, Camden 08003, New Jersey, USA

## Abstract

Since their discovery, human induced pluripotent stem cells (hiPSCs) have been instrumental in biomedical research, particularly in the fields of disease modelling, drug screening and regenerative therapies. Their use has significantly increased over recent years driven by the ability of hiPSCs to provide differentiated cell models without requiring embryonic stem cells. Furthermore, the transition from integrating to non-integrating reprogramming methodologies has contributed to the increase in utilisation. This shift minimises the risk of genomic alterations, enhancing the safety and reliability of hiPSCs. However, the factors that contribute to reprogramming success are still not well understood. In this study, we conducted a comparative analysis of the most prevalent non-integrating reprogramming methods across a range of starting source materials to assess their impact on reprogramming success rates. We found that while source material does not significantly impact success rates, the Sendai virus reprogramming method yields significantly higher success rates relative to the episomal reprogramming method. Our findings offer important insights from a biobanking perspective, for which long-term reliability, integrity and reproducibility of hiPSCs are crucial.

## 1. Introduction

In 2006 the first induced pluripotent stem cells (iPSCs) were developed [1], and 1 year later human fibroblasts were successfully reprogrammed [2, 3] into human iPSCs (hiPSCs). Despite the numerous advantages of hiPSCs, early retroviral and lentiviral reprogramming methods gave rise to significant concerns regarding the therapeutic potential of these cells, largely due to insertional mutagenesis and residual expression of reprogramming factors that can be caused by the integration of the virus into the host genome [4–8].

Genomic instability resulting from the earliest reprogramming methods encouraged the development of non-integrating techniques. Alternative methods include the use of adenovirus, Sendai virus (SeV) and episomal vectors as well as plasmids, miRNA, mRNA, small molecules and protein transduction [9–13]. Among them, SeV and episomal reprogramming methods are the most commonly used due to ease of manipulation and relative efficiency compared to other reprogramming methods [14–16]. Indeed, it has been shown that these two non-integrating methods have a significantly lower number of copy number variants (CNVs), single nucleotide polymorphisms (SNPs) and chromosomal mosaicism relative to the lentiviral integrating method [14]. Interestingly, a comparison of the integration-free reprogramming methods does not offer a clear conclusion regarding which method yields the highest success rates. For instance, in one study [15] examining the efficiency and success rates of non-integrating reprogramming methods, it was noted that although the SeV method showed greater efficiency than the episomal system, there was no significant difference in success rates between the two methods.

The National Institute of General Medical Sciences (NIGMS) Human Genetic Cell Repository (NIGMS Repository) is a highly unique resource of biospecimens collected from patients diagnosed with genetic diseases and apparently healthy individuals. NIGMS Repository samples are annotated with rich clinical, demographic and phenotypic data and are available to scientists worldwide (https://www.coriell.org/1/NIGMS). Importantly, the NIGMS Repository also contains a growing collection of iPSCs [17]. From a biobanking perspective, generating high-quality hiPSCs with low batch-to-batch variation is important to maximise consistency and reproducibility in basic research, disease modelling, drug screening and translational studies. Achieving this goal requires standardised reprogramming protocols and stringent quality control measures at every stage of the iPSC generation and expansion process.

In the stem cell biology field, there is a well-established preference for non-integrating reprogramming methods over lentiviral and retroviral approaches due to their reduced risk of genomic integration [14]; however, a comprehensive investigation of non-integrating methods from a biobanking perspective is currently lacking. Furthermore, to the best of our knowledge, there has not been any exploration into how the source of the starting material might impact the success rate of reprogramming. To address these questions, we conducted a systematic comparison of the most prevalent in-house reprogramming methods—episomal and SeV reprogramming. Additionally, we assessed the impact of source material by comparing reprogramming success rates across common starting cell types, including fibroblasts, lymphoblastoid cell lines (LCLs) and peripheral blood mononuclear cells (PBMCs). Overall, our study contributes to a better understanding of the direct and indirect factors influencing the success rates of the reprogramming process, thereby, maximising the research potential of NIGMS Repository iPSCs.

## 2. Materials and Methods

### 2.1. Cell Culture

Cells were thawed by gentle twirling in 37°C. Afterwards, the vial containing the cells was dried, dowsed with 70% ethanol and wiped dry again prior to placing it into the hood. In the next step, 50 µL of the thawed cells were placed onto a blood agar bacteriological plate and streaked using a pipette tip. The rest of the cells were added dropwise to the room temperature prewarmed growth medium (mTeSR1 complete medium). The cells were centrifuged at 1100 rpm for 3 min and the pellet was resuspended within a 2 mL growth medium containing 10 µM Y-27632 (ROCK inhibitor). The ROCK inhibitor was removed 20–24 h post-thaw. Mouse embryonic fibroblast (MEF) media or Matrigel solution were removed from the plate and the cell suspension was added to the well and equally distributed within the well by moving the plate in opposite directions. The plate was placed in an incubator and the media was exchanged daily with a growth medium equilibrated to room temperature. One day per week, “double feeding” was performed by exchanging the growth medium with 3 mL of fresh medium and not feeding the cells on the following day.

In general, the cells were passaged when reaching 75%–85% confluency and before the colonies became too large or too dense and began differentiating in the centre. In brief, spent media was removed, the cells were rinsed once with PBS and 1 mL of enzyme solution was added to the well for the following incubation times: Versene: 2 min at room temperature and ReLeSR: 5–7 min of dry incubation at 37°C. When ReLeSR was used, the colonies were detached by the addition of 1 mL of mTeSR1 and manual tapping on the sides of the plate. The cell aggregates were collected and seeded at multiple wells at optimal density. When Versene was used, 2 mL of growth medium was added for a wash. Afterwards, the cells were scraped with a cell scraper, evenly distributed into the wells, and placed in an incubator set at 37°C and 5% CO_2_. Percentage of differentiation, removal method and split ratio were recorded. Cell lines were maintained and passaged until the cells had been expanded to a minimum of nine, preferably 12 wells of a six-well plate for the five-vial master bank (MB) freeze followed by five six-well plates for a 26-vial distribution bank (DB) freeze.

By the second passage post-recovery, one well of the six-well plate was submitted for mycoplasma detection by placing 0.5 mL spent medium and 0.5 mL cell suspension into a mycoplasma test glass vial.

Cells were cryopreserved when they appeared healthy, 75%–85% confluent and undifferentiated. The cell number was in the range of 1 × 10^6^–2 × 10^6^ per freezing vial. For feeder-grown iPSCs, 90% ESC-qualified FBS was used with 10% DMSO. For feeder-free iPSCs, 90% KOSR was used with 10% DMSO. The components were mixed together on the day of the freeze and were filter-sterilized. MB freeze was performed when the cells were expanded to a minimum of nine wells. At this stage, the cell pellet was collected for identity testing (short tandem repeat (STR) comparison), a T25 flask was set up for karyotyping and 2.5 mL spent medium and 0.5 mL cell suspension was removed for sterility testing. At the stage of DB freeze, one well was used for alkaline phosphatase (AP) staining. In general, for freezing, the cells were dissociated enzymatically, and the detached cell aggregates were centrifuged at 1100 rpm for 3 min at room temperature. Afterwards, the cells were resuspended in 1 mL of freezing medium per each well of a six-well plate, making sure that each vial had 1−2 × 10^6^ cells. The vials were transported on ice or cold blocks to the cryogenics department where cells were frozen in an electronically controlled freeze chamber.

### 2.2. Non-Integrating Reprogramming Methods

#### 2.2.1. Episomal Reprogramming

**Figure 1:**
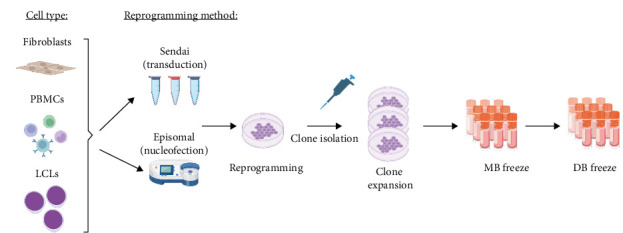
Schematic representation of the reprogramming process. DB, distribution bank; LCLs, lymphoblastoid cell lines; MB, master bank; PBMCs, peripheral blood mononuclear cells.

For episomal reprogramming of the LCLs and fibroblasts with OriP/EBNA1 episomal vectors expressing hOCT3/4 with sh-p53, hSOX2, hKLF4, hL-MYC, LIN28 and EGFP, the cells were nucleofected with Amaxa Nucleofector II device (Lonza) using U-015 (LCLs) and U-023 (fibroblasts) programmes and maintained at 37°C, 5% CO_2_ and 5% O_2_ incubator. The cells were fed every other day post-nucleofection. Nucleofection efficiency was assessed by monitoring GFP-positive cells. On days 6–7 after nucleofection, the transfected cells were replated. After 1–2 additional weeks, at least 24 clones were selected manually for further expansion. The clones were fed daily until enzymatic passaging and expansion for MB and DB (Figure 1).

#### 2.2.2. SeV Reprogramming

For reprogramming of the fibroblasts and PBMCs by transduction with four SeV vectors expressing hOCT4, hSOX2, hKLF4, hC-MYC and EmGFP, the CytoTune Sendai Reprogramming Kit (Thermo Fisher Scientific) was used. Twenty-four hours after transduction, the medium was refreshed with fresh medium, and the cells were cultured for approximately 6 additional days with medium exchanged every other day. Transduction efficiency was estimated by examination of GFP-positive cells. Approximately 7 days following the transduction for fibroblasts and 3 days following transduction for PBMCs, the cells were ready to be harvested and replated. After an additional 2–3 weeks, colonies reached the appropriate size for transfer, and at this stage at least 24 colonies were manually picked. Approximately at passage 10, several cryovials for a MB were cryopreserved followed by expansion and cryopreservation of vials for DB (Figure 1).

### 2.3. Quality Control

To ensure iPSC reliability and reproducibility, several quality control measures were applied to each reprogrammed cell line (Table 1).

### 2.4. Microbial Sterility Check

Upon thawing of any cell line, inoculation of sheep's blood agar plates was performed. Briefly, upon first thaw, 50 µL of cell suspension was streaked on one blood agar plate and incubated at 37°C. At the MB freeze of external hiPSCs and at the freeze recovery of QC of hiPSCs DB vials, inoculation of broth tubes was conducted in addition to sample submission for mycoplasma testing.

### 2.5. Surface Antigen (SA) Staining

When cells became at least 75% confluent, the medium was removed and they were rinsed with PBS. Afterwards, the cells were enzymatically dissociated, triturated to achieve single-cell suspension, centrifuged and stained for stage-specific embryonic antigen 4 (SSEA4) which is expressed on the surface of undifferentiated hiPSCs. The cells were resuspended in FACs staining buffer and analysed using the MACSQuant Flow Cytometer (Miltyeni Biotec).

### 2.6. Embryoid Body (EB) Formation

To test differentiation potential, the capacity of iPSCs to form EBs was examined. Briefly, when the colonies reached an appropriate size for EB set up, the cells were dissociated using Versene, TrypLE Express or Collagenase B containing DNase1. The cells were centrifuged at 800 rpm and mixed with hEB medium supplemented with DNAse1. The cell suspension was transferred to low attachment plates while being evenly distributed to the same number of wells that were initially harvested. The cells were incubated at 5% O_2_ incubator at 37°C. After 48 h the medium was changed by collecting the EBs in a 50 mL conical tube, centrifugation at 800 rpm for 3 min and supplementation with new medium. The medium was changed in a similar manner every other day, until harvesting of the EBs on day 10. For harvesting, the EBs were washed in PBS, resuspended in RLT lysis buffer (Qiagen) and processed for RNA extraction.

### 2.7. Real Time PCR (RT-PCR)

To measure gene expression for confirmation of EB formation quantitative RT-PCR was performed. Additionally, between passage 15 and 20 of newly reprogrammed hiPSCs, cDNA samples are analysed by RT-PCR for identification of episomal reprogramming factors integration. Briefly, RNA was extracted according to the manufacturer's instructions using QiaCube, RNAeasy mini kit or RNAeasy micro kit. Afterwards, cDNA was synthesised using the high-capacity cDNA reverse transcriptase kit (Applied Biosystems). Gene expression was evaluated with a commercially available (Applied Biosystems) TaqMan Assay using on demand primers (OCT4: hs00742896_s1; SOX2: hs00602736_s1; NANOG: hs02387400_g1; GDF3: hs0020998_m1; REXO1: hs00381890_m1; PAX6: hs00240871_m1; NES: hs00707120_s1; TP63: hs00978340_m1; KRT14: hs00265033_m1; NOG: hs00271352_s1; T: hs00610080_m1; RUNX1: hs01021970_m1; DES: hs0015729_m1; PECAM1: hs00169777_m1; TAL1: hs01097987_m1; AFP: hs00173490_m1; SOX17: hs00751752_s1; FOXA2: hs00232764_m1; SOX7: hs00846731_s1; GAPDH: hs002667705_g1). Ct values were adjusted to the endogenous housekeeping gene GAPDH.

### 2.8. AP Surface Staining

We used AP staining as a biomarker for identifying stem cells [18]. When cells reached at least 40%–50% confluency, they were stained for AP using the StemTAG Alkaline Phosphatase Staining Kit (Cell Biolabs). In brief, the medium was aspirated and the cells were rinsed with 2 mL of rinsing buffer. Afterwards, the cells were incubated for 2 min with 1 mL of fixing solution, washed twice with rinsing buffer and incubated in the dark with StemTAG AP Staining Solution for 15–30 min. The cells were washed twice, supplemented with PBS and visualised at 4x magnification for subsequent determination of the percentage of stained and undifferentiated colonies.

### 2.9. Statistical Analysis

To assess the potential contributions of reprogramming method and source cell type on reprogramming success (Table 2 and Supporting Information: Table S1), we employed logistic regression using the glm function in R. We first independently assessed each factor variable (logistic regression modelling results for reprogramming method and source material are summarised in Supporting Information: Table S2, S3). We additionally performed *χ*^2^ tests to assess the independent effect of the reprogramming method and source material on success rate. However, given that these variables are not completely independent of each other, we additionally used multivariate logistic regression to model both variables together in a single model with success rate as the dependent variable (Table 3).

## 3. Results

Despite stringent quality control procedures, not all iPSCs pass quality control assessments to qualify for long-term distribution to the research community. To better understand the factors contributing to iPSC quality control success, we examined whether the reprogramming method and starting source material influence reprogramming success rate (Table 3 and Supporting Information: Table S2, S3). To this end, success was defined as a cell line that has passed all quality control steps necessary for the eventual inclusion of the cell line into the NIGMS Repository housed at Coriell. Importantly, these quality control measures are in alignment with the standards for human stem cell use in research outlined by the International Society for Stem Cell Research [19].

**Figure 2:**
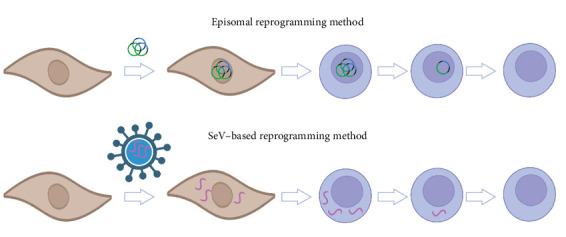
Schematic representation of episomal and Sendai virus (SeV) reprogramming methods. In episomal reprogramming, sustained expression of reprogramming factors is achieved through Epstein–Barr virus–derived sequences, facilitating the replication of exogenous DNA in the nucleus of dividing cells, thereby, ensuring the continuous expression of these factors throughout the reprogramming process. In SeV reprogramming, viral particles serve as vectors to deliver replication-competent RNAs encoding the original set of reprogramming factors into target cells, facilitating the expression of essential reprogramming factors crucial for cell fate conversion. Since SeV is an RNA virus, it bypasses the need to enter the nucleus for transcription, thus, eliminating the possibility of transgene integration into the host genome.

First, we evaluated the effect of two reprogramming methods on the reprogramming success rate—the episomal reprogramming method and the SeV reprogramming method (Figure 2). Episomal vectors, consisting of Epstein–Barr virus–derived components, offer a non-integrative means of delivering reprogramming factors into somatic cells, enabling transient expression and reducing the risk of insertional mutagenesis associated with retroviruses and lentiviruses [20]. Similarly, SeV, characterised by its negative single-stranded RNA, efficiently transports genes into various somatic cells, offering another non-integrative vector option.

**Figure 3:**
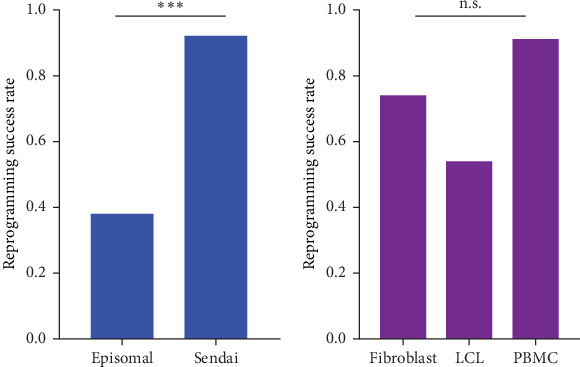
Success rate comparison of non-integrating methods. (A) Reprogramming success rate comparison between episomal (*n* = 26) and Sendai virus (*n* = 50) reprogramming methods for human fibroblast, lymphoblastoid cell line (LCL) and peripheral blood mononuclear cell (PBMC) experiments. Success was defined as a cell line that has passed all quality control steps necessary for the eventual inclusion of the cell line in Coriell's online catalog. (B) Reprogramming success rate comparison between different source materials: fibroblasts (*n* = 54), LCLs (*n* = 11), PBMCs (*n* = 11). *⁣*^*∗∗∗*^*p* < 0.001; n.s. = not significant.

**Figure 4:**
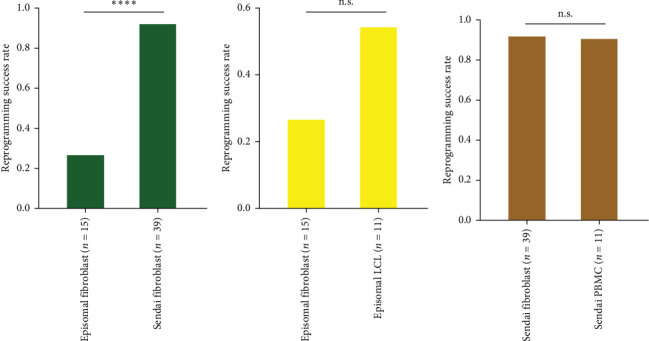
Comparison of reprogramming success rates between different reprogramming methods and source materials. (A) Reprogramming success rates for episomal fibroblasts (*n* = 15) and Sendai virus (SeV)-transduced fibroblasts (*n* = 39). (B) Reprogramming success rates for episomal fibroblasts (*n* = 15) and episomal lymphoblastoid cell lines (LCLs, *n* = 11). (C) Reprogramming success rates for SeV-transduced fibroblasts (*n* = 39) and SeV-transduced peripheral blood mononuclear cells (PBMCs, *n* = 11). A *χ*^2^ test was performed to compare the success rates between the groups. *⁣*^*∗∗∗∗*^*p* < 0.0001; n.s. = not significant.

We reviewed a total of 50 cell lines reprogrammed with the SeV method and 26 cell lines reprogrammed with the episomal method (Table 2). Descriptions of the cell lines used in the study are presented in Supporting Information: Table S1. Our results demonstrate that the SeV reprogramming method leads to a significantly higher reprogramming success rate (92.0%) compared to the episomal reprogramming method (38.5%) regardless of source material (Figure 3A and Table 3). To ensure that this result is robust to source material heterogeneity, we performed a more focused comparison of reprogrammed fibroblasts (the single source material reprogrammed with both methods). We found that consistent with the multivariate model results, fibroblast cell lines reprogrammed using the SeV method (*n* = 39) demonstrate a significantly higher success rate (92.3%) compared to those reprogrammed with the episomal method (*n* = 15; 26.7%), (*χ*^2^ = 21.008, *p*  < 0.000004574; Figure 4A).

Through the expression of specific transcription factors, hiPSCs can be reprogrammed from various dividing somatic cell types [21]. While fibroblasts were the first cell type utilised for reprogramming and remain one of the most widely used sources for hiPSC generation, other cell types such as keratinocytes and adipose-derived cells have also been successfully employed for hiPSC generation. Among the alternative cell sources, whole blood has become increasingly favoured for its minimally invasive collection procedure [21]. As no comparison has been conducted to date, among the various cell sources, we compared three different cell types utilised for reprogramming (Table 2). Namely, we compared reprogramming success rates for fibroblasts (*n* = 54; 74.1%), LCLs (*n* = 11; 54.5%) and PBMCs (*n* = 11; 90.1%). However, we found no significant difference in the reprogramming success rates among the different source material cell types (Figure 3B and Table 3). Consistent with the multivariate analysis, we found no significant difference in our more focused comparisons (Figure 4B,C). More specifically, there is no significant episomal reprogramming success rate difference between source material (fibroblast 26.7% vs. LCL 54.5%; *χ*^2^ = 1.0725, *p*=0.3; Figure 4B). Similarly, there is no significant Sendai reprogramming success rate difference between source material (fibroblast 92.3% vs. PBMC 90.1%; *χ*^2^ = 9.9969 × 10^−30^, *p*=1.0; Figure 4C). Therefore, all of our source material analyses consistently show that source material does not significantly influence reprogramming success outcomes.

## 4. Discussion

In this work, we compared the two commonly used non-integrating reprogramming methods—the episomal and SeV methods (Figure 2). They both offer a distinct advantage over lenti/retroviral reprogramming due to their lack of genomic integration, potentially resulting in transgene-free and genomically stable iPSCs.

Episomal reprogramming, pioneered by the Thomson Group in 2009 [22], has since been refined by multiple research teams. The modifications have often involved the addition of reprogramming factors to the conventional Yamanaka factors, tailored with specific combinations of promoters to boost reprogramming efficiencies. For instance, various studies have investigated the potential benefits of inhibiting the P53 tumour suppressor and genome stability pathway during episomal reprogramming [23]. Indeed, increased efficiencies were observed when employing several strategies to inactivate the P53 pathway, such as co-expressing p53 shRNA [10, 24]. In addition, since different groups have shown that transient enhancement of EBNA1 expression levels at the beginning of the reprogramming process increases the efficiency of reprogramming [25–27], we chose to use OriP/EBNA1 episomal vectors that express sh-p53 among other reprogramming factors. The advantage of the episomal approach is that compared to viral methods, the construction and production of the plasmid are relatively simple [28]. In addition, it has been demonstrated that in early passages, episomally reprogrammed iPSCs become transgene-free [10, 22]. Nevertheless, the concern of potential exogenous plasmid DNA presence persists, demanding a careful validation of the absence of these sequences [28]. Another disadvantage of the method is that compared to other non-integrating methods, it yields cells with slightly increased levels of genetic aberrations such as increased aneuploidy rates [15]. Consistent with these reports, we have found that episomal reprogramming yields lower success rates compared to the SeV reprogramming method (Figure 3A).

SeV is an RNA virus that infects the cells by binding to the expressed sialic acid residues. Even though it does not evade the human innate system, the virus still efficiently infects human cells, without being pathogenic [29]. SeV based reprogramming was first demonstrated by the Hasegawa laboratory which utilised it for reprogramming human fibroblasts [30]. In the current study we have employed the CytoTune SeV approach due to its high reprogramming efficiency and single transduction process [31].

A previous study [15] compared the efficiency and success rates of non-integrating reprogramming methods, including mRNA–based reprogramming, SeV–based system and episomal system methods. The results of the study indicate that even though the SeV method is more efficient than the episomal system; no difference in the success rates between the two methods was found when success was defined as yielding at least three colonies. In the current study, we defined success more comprehensively as the subset of iPSC lines that passed all quality control steps directly relevant to short-term reprogramming success as well as all of the direct and indirect factors that contribute to the long-term stability, viability, sterility and quality of the iPSC lines, consistent with International Society for Stem Cell Research standards [19]. From the iPSC biobanking perspective that prioritises the iPSC line consistency and continuity to support large-scale distribution activities over time, taking into account all quality control steps is crucial. In the current study, we included AP staining, viral genome and transgene detection, SA expression of stem cell markers and cytogenomics as direct measures of reprogramming success. We additionally included several indirect measures of long-term iPSC culture success. We used post-thaw variability to assess the stress of viral integration or chemical treatment during reprogramming, which could affect cellular resilience; we included two measures of sterility and an additional measure of cell line authenticity (via STR) to ensure against contamination and cross-contamination, respectively, which can be introduced through the lengthy reprogramming process. Successful reprogramming must generate iPSC lines that are both biologically valid and practically usable over time. Our success definition meets or exceeds industry standards [19], ensuring only fully validated, repository-ready cell lines pass quality control. These comprehensive quality measures safeguard research reproducibility, ultimately serving the research community more effectively.

Similarly to the aforementioned research, other comparative studies were not able to detect consistent and significant differences between episomal and SeV methods of reprogramming [14, 32]. In these cases, the reduced number of cell lines employed in the studies may account for the negative findings.

In addition to comparing the two reprogramming methods, we have examined the effect of different source materials on the success of reprogramming. To our knowledge, this comparison has not been conducted previously. We have found no significant difference between the different source materials and their effect on the reprogramming success rate. Namely, the success rate was similar between fibroblasts, PBMCs and LCLs (Figure 3B). Overall, our results emphasise the importance of choosing the most appropriate reprogramming method for the generation of consistently successful hiPSC cell lines.

## 5. Conclusions

In summary, our study demonstrates that while the source material does not significantly impact reprogramming success rates, the SeV method is notably more effective than the episomal approach, providing crucial insights for the reliable and reproducible generation of high-quality hiPSCs in biobanking.

## Figures and Tables

**Table 1:** Quality control measures applied to each cell line.

Test description	Test method	Test specification
Post-thaw cell viability	Colony doubling	Colony formation and diameter doubling within 5 days
Sterility	Growth on agar and broth	Negative
Mycoplasma	qRT-PCR	Negative
Alkaline phosphatase (AP) staining	Cell staining	>80% cells with positive staining
Identity match	Short tandem repeat (STR)	Match parental profile
Detection of viral genome and transgene	qRT-PCR using specific primers	No detection of Sendai virus (SeV) genome or transgenes
Surface antigen (SA) expression of stem cell markers	Immunostaining and flow cytometric analysis	>80% expression of stage-specific embryonic antigen 4 (SSEA4)
Differentiation potential	Embryoid body (EB) formation and gene expression	Minimum of one gene per germ layer expressed twofold or higher
Cytogenomics	G-banding	46 XX; 46 XY

**Table 2:** The number of cell lines used for success rate comparison by method of reprogramming and by source material.

Source/reprogramming method	Sendai	Episomal
Fibroblasts	39	15
Lymphoblastoid cell lines (LCLs)	0	11
Peripheral blood mononuclear cells (PBMCs)	11	0

**Table 3:** Multivariate logistic regression results for reprogramming method and source material.

	Estimate	Standard error	*Z* value	*p* value
Intercept	−1.0116	0.583	−1.732	0.083
Reprogramming method	3.496	0.837	4.173	3.00e−05
Source material (lymphoblastoid cell line (LCL))	1.193	0.841	1.419	0.155
Source material (peripheral blood mononuclear cell (PBMC))	−0.182	1.208	−0.150	0.880

## Data Availability

The data that support the findings of this study are available from the corresponding author upon reasonable request.

## Supporting Information

Supporting Information The file contains a detailed description of the cell lines used in the study as well as additional statistical results that include logistic regression modeling results for the reprogramming method and source material.
