# Supplementary material for: Human iPSC Reprogramming Success: The Impact of Approaches and Source Materials
Source: Stem Cells Int. 2025 Jan 16;2025:2223645. doi: 10.1155/sci/2223645 (PMC11756937; doi:10.1155/sci/2223645)
Supplement: Supporting Information — The file contains a detailed description of the cell lines used in the study as well as additional statistical results that include logistic regression modeling results for the reprogramming method and source material. [file 2223645.f1.docx]

Supplementary materials:

| Sample ID | Diagnosis | Success  /failure | method of reprogramming | source |
| --- | --- | --- | --- | --- |
| GM22870 | NIEMANN-PICK DISEASE | Failure | episomal | fibroblast |
| GM16684 | XERODERMA PIGMENTOSUM | Failure | episomal | fibroblast |
| GM07371 | PEROXISOME BIOGENESIS DISORDER | Failure | episomal | fibroblast |
| GM04025 | FRAGILE X MENTAL RETARDATION SYNDROME | Failure | episomal | LCL |
| GM13411 | LEIGH SYNDROME | Failure | Sendai | fibroblast |
| GM12878 | CEPH/UTAH PEDIGREE | Failure | episomal | LCL |
| GM25313 | MUSCULAR DYSTROPHY, DUCHENNE TYPE | Success | episomal | fibroblast |
| GM25318 | TUBEROUS SCLEROSIS 2 | Success | episomal | fibroblast |
| GM25323 | DIGEORGE SYNDROME | Failure | episomal | fibroblast |
| GM25532 | MARFAN SYNDROME | Failure | episomal | fibroblast |
| GM25541 | LESCH-NYHAN SYNDROME | Success | episomal | fibroblast |
| GM25545 | GAUCHER DISEASE | Failure | episomal | fibroblast |
| GM25553 | GLYCOGEN STORAGE DISEASE II | Failure | episomal | fibroblast |
| GM25558 | ADRENOLEUKODYSTROPHY | Failure | episomal | fibroblast |
| GM25864 | NIEMANN-PICK DISEASE, TYPE C1 | Success | Sendai | fibroblast |
| GM25942 | NIEMANN-PICK DISEASE, TYPE C1 | Failure | episomal | fibroblast |
| GM25967 | EHLERS-DANLOS SYNDROME | Failure | episomal | fibroblast |
| GM25971 | MUCOPOLYSACCHARIDOSIS TYPE VII | Failure | episomal | fibroblast |
| GM26017 | EPIDERMOLYSIS BULLOSA | Success | Sendai | fibroblast |
| GM26025 | MILLER-DIEKER LISSENCEPHALY SYNDROME | Success | Sendai | fibroblast |
| GM26029 | KEARNS-SAYRE SYNDROME | Success | Sendai | fibroblast |
| GM26077 | PERSONAL GENOME PROJECT | Success | episomal | LCL |
| GM26085 | PERSONAL GENOME PROJECT | Failure | episomal | LCL |
| GM26105 | PERSONAL GENOME PROJECT | Success | episomal | LCL |
| GM26107 | PERSONAL GENOME PROJECT | Success | episomal | LCL |
| GM26644 | KRABBE DISEASE | Success | Sendai | fibroblast |
| GM26650 | CHOROIDEREMIA | Success | Sendai | fibroblast |
| GM26656 | HURLER SYNDROME | Success | Sendai | fibroblast |
| GM26663 | CHOROIDEREMIA | Success | Sendai | fibroblast |
| GM27162 | MUCOPOLYSACCHARIDOSIS TYPE IIIA | Success | Sendai | fibroblast |
| GM27175 | CEROID LIPOFUSCINOSIS | Success | episomal | LCL |
| GM27212 | CEPH/UTAH PEDIGREE | Failure | episomal | LCL |
| GM27270 | PITT-HOPKINS SYNDROME | Success | Sendai | fibroblast |
| GM27291 | VICI SYNDROME | Success | Sendai | fibroblast |
| GM27327 | FACIOSCAPULOHUMERAL MUSCULAR DYSTROPHY 1 | Success | episomal | LCL |
| GM27380 | ASYMPTOMATIC OR UNDIAGNOSED AND GENETICALLY RELATED TO AN AFFECTED INDIVIDUAL | Success | Sendai | fibroblast |
| GM27437 | RETT SYNDROME | Success | Sendai | fibroblast |
| GM27578 | PHENYLKETONURIA | Success | Sendai | fibroblast |
| GM27716 | APPARENTLY HEALTHY INDIVIDUAL | Success | Sendai | fibroblast |
| GM27730 | PERSONAL GENOME PROJECT | Success | Sendai | PBMC |
| GM27855 | CEREBRAL CREATINE DEFICIENCY SYNDROME 1 | Success | Sendai | fibroblast |
| GM27860 | MENTAL RETARDATION, AUTOSOMAL DOMINANT 40 | Success | Sendai | fibroblast |
| GM27918 | SMITH-MAGENIS SYNDROME | Success | Sendai | fibroblast |
| GM27925 | OSTEOGENESIS IMPERFECTA, TYPE IV | Success | Sendai | fibroblast |
| GM27929 | ACUTE LYMPHOCYTIC LEUKEMIA | Success | Sendai | PBMC |
| GM27939 | MUSCULAR DYSTROPHY-DYSTROGLYCANOPATHY | Success | Sendai | fibroblast |
| GM27957 | MENTAL RETARDATION, AUTOSOMAL DOMINANT 5 | Success | Sendai | PBMC |
| GM27978 | MENTAL RETARDATION, AUTOSOMAL DOMINANT 40 | Success | Sendai | fibroblast |
| GM28249 | CANAVAN DISEASE | Success | Sendai | fibroblast |
| GM28258 | TAY-SACHS DISEASE | Failure | Sendai | fibroblast |
| GM28259 | TAY-SACHS DISEASE | Success | Sendai | fibroblast |
| GM24559 | DYSTROPHIA MYOTONICA 1 | Success | episomal | fibroblast |
| GM28374 | NEUROAXONAL DYSTROPHY | Success | Sendai | fibroblast |
| GM28207 | SCHUURS-HOEIJMAKERS SYNDROME | Failure | Sendai | PBMC |
| GM28339 | SCHUURS-HOEIJMAKERS SYNDROME | Success | Sendai | fibroblast |
| GM28622 | GLYCOGEN STORAGE DISEASE II | Success | Sendai | fibroblast |
| GM28589 | CEREBRAL CREATINE DEFICIENCY SYNDROME 3 | Success | Sendai | PBMC |
| GM28862 | LEIGH SYNDROME | Success | Sendai | fibroblast |
| GM28907 | APPARENTLY HEALTHY INDIVIDUAL | Success | Sendai | fibroblast |
| GM28891 | SCHUURS-HOEIJMAKERS SYNDROME | Success | Sendai | fibroblast |
| GM28603 | WIEACKER-WOLFF SYNDROME | Success | Sendai | PBMC |
| GM28937 | CEREBRAL CREATINE DEFICIENCY SYNDROME 1 | Success | Sendai | PBMC |
| GM28947 | HELSMOORTEL-VAN DER AA SYNDROME | Success | Sendai | PBMC |
| GM28959 | TUBEROUS SCLEROSIS 1; | Success | Sendai | fibroblast |
| GM28955 | TUBEROUS SCLEROSIS 2 | Success | Sendai | fibroblast |
| GM28930 | ISOGENIC CONTROL | Success | Sendai | fibroblast |
| GM28975 | MYOPATHY, DISTAL, 5 | Success | Sendai | PBMC |
| GM29085 | PERSONAL GENOME PROJECT | Success | episomal | LCL |
| GM29090 | PERSONAL GENOME PROJECT | Success | Sendai | fibroblast |
| GM29087 | PERSONAL GENOME PROJECT | Success | Sendai | fibroblast |
| GM28982 | MYOPATHY, DISTAL, 5 | Success | Sendai | PBMC |
| GM29132 | LEIGH SYNDROME | Success | Sendai | fibroblast |
| GM28936 | CEREBRAL CREATINE DEFICIENCY SYNDROME 2 | Success | Sendai | PBMC |
| GM29080 | LEIGH SYNDROME | Failure | Sendai | fibroblast |
| GM29077 | PERSONAL GENOME PROJECT | Failure | episomal | LCL |
| GM28978 | APPARENTLY HEALTHY INDIVIDUAL | Success | Sendai | fibroblast |

**Table S1: Description of the cell lines used in the study.**

|  | Estimate | Std. Error | Z value | Pr(>\|z\|) |
| --- | --- | --- | --- | --- |
| Intercept | -0.4700 | 0.4031 | -1.166 | 0.244 |
| Sendai | 2.9124 | 0.6590 | 4.420 | 9.89e-06 |

**Table S2: Logistic regression modeling results for reprogramming method.**

|  | Estimate | Std. Error | Z value | Pr(>\|z\|) |
| --- | --- | --- | --- | --- |
| Intercept | 1.0498 | 0.3105 | 3.381 | 0.000723 |
| LCL | -0.8675 | 0.6805 | -1.275 | 0.202388 |
| PBMC | 1.2528 | 1.0636 | 1.146 | 0.251989 |

**Table S3: Logistic regression modeling results for source material.**
